# Adjuvant capecitabine-containing chemotherapy benefit and homologous recombination deficiency in early-stage triple-negative breast cancer patients

**DOI:** 10.1038/s41416-022-01711-y

**Published:** 2022-02-05

**Authors:** Leonora W. de Boo, Katarzyna Jóźwiak, Heikki Joensuu, Henrik Lindman, Susanna Lauttia, Mark Opdam, Charlaine van Steenis, Wim Brugman, Roelof J. C. Kluin, Philip C. Schouten, Marleen Kok, Petra M. Nederlof, Michael Hauptmann, Sabine C. Linn

**Affiliations:** 1 Division of Molecular Pathology, The Netherlands Cancer Institute, Amsterdam, The Netherlands; 2 Institute of Biostatistics and Registry Research, Brandenburg Medical School Theodor Fontane, Neuruppin, Germany; 3 Department of Epidemiology and Biostatistics, The Netherlands Cancer Institute, Amsterdam, The Netherlands; 4 Department of Oncology, Helsinki University Hospital and University of Helsinki, Helsinki, Finland; 5 Department of Immunology, Genetics and Pathology, Uppsala University Hospital, Uppsala, Sweden; 6 Laboratory of Molecular Oncology, Biomedicum Helsinki, University of Helsinki, Helsinki, Finland; 7 Genomics Core Facility, The Netherlands Cancer Institute, Amsterdam, The Netherlands; 8 Department of Medical Oncology, The Netherlands Cancer Institute, Amsterdam, The Netherlands; 9 Division of Tumor Biology & Immunology, The Netherlands Cancer Institute, Amsterdam, The Netherlands; 10 Department of Pathology, The Netherlands Cancer Institute - Antoni van Leeuwenhoek Hospital, Amsterdam, The Netherlands; 11 Department of Pathology, University Medical Centre, Utrecht, The Netherlands

**Keywords:** Breast cancer, Translational research, Predictive markers, Breast cancer

## Abstract

**Background:**

The addition of adjuvant capecitabine to standard chemotherapy of early-stage triple-negative breast cancer (TNBC) patients has improved survival in a few randomised trials and in meta-analyses. However, many patients did not benefit. We evaluated the *BRCA1*-like DNA copy number signature, indicative of homologous recombination deficiency, as a predictive biomarker for capecitabine benefit in the TNBC subgroup of the FinXX trial.

**Methods:**

Early-stage TNBC patients were randomised between adjuvant capecitabine-containing (TX + CEX: capecitabine-docetaxel, followed by cyclophosphamide-epirubicin-capecitabine) and conventional chemotherapy (T + CEF: docetaxel, followed by cyclophosphamide-epirubicin-fluorouracil). Tumour *BRCA1*-like status was determined on low-coverage, whole genome next-generation sequencing data using an established DNA comparative genomic hybridisation algorithm.

**Results:**

For 129/202 (63.9%) patients the *BRCA1*-like status could be determined, mostly due to lack of tissue. During a median follow-up of 10.7 years, 35 recurrences and 32 deaths occurred. Addition of capecitabine appears to improve recurrence-free survival more among 61 (47.3%) patients with non-*BRCA1*-like tumours (HR 0.23, 95% CI 0.08–0.70) compared to 68 (52.7%) patients with *BRCA1*-like tumours (HR 0.66, 95% CI 0.24–1.81) (P-interaction = 0.17).

**Conclusion:**

Based on our data, patients with non-*BRCA1*-like TNBC appear to benefit from the addition of capecitabine to adjuvant chemotherapy. Patients with *BRCA1*-like TNBC may also benefit. Additional research is needed to define the subgroup within *BRCA1*-like TNBC patients who may not benefit from adjuvant capecitabine.

## Background

Triple-negative breast cancer (TNBC) accounts for 10–20% of all breast cancers and is associated with a high risk of early recurrence and poor survival once metastasised [1, 2]. Trials evaluating escalation of adjuvant treatment are emerging [3, 4]. A recent meta-analysis including 3854 early-stage TNBC patients showed that adjuvant capecitabine following or added to standard neoadjuvant anthracycline- and taxane-based therapy substantially improved survival [5–7]. Hence, this approach has been incorporated in current national and international guidelines [8–10]. Capecitabine is a prodrug of 5-fluoruouracil and belongs to the class of antimetabolites. It shows cytotoxic activity through the inhibition of thymidylate synthase and the incorporation of its metabolites into DNA and RNA [11]. Although treatment with adjuvant capecitabine is promising in HER2-negative patients, the benefit is limited to a subgroup given the absolute disease-free survival benefit of only 8.9% at 3 years [5]. Thus, there is an unmet clinical need to identify the subgroup of patients that will benefit. To our knowledge, the only study searching for biomarkers that predict capecitabine benefit failed to identify a predictive marker in an 800-gene expression explorative analysis [12]. Furthermore, an absolute survival benefit comparable to adjuvant capecitabine was recently observed in HER2-negative patients treated with adjuvant olaparib in the OlympiA trial [13]. Olaparib, a polyadenosine 5’diphosphoribose polymerase (PARP) inhibitor, resulted in an absolute invasive disease-free survival (IDFS) benefit of 8.8% at 3 years for patients with early-stage, high-risk, germline *BRCA*-mutated, HER2 negative breast cancer. However, these patients were not treated according to the current clinical practice of a capecitabine-containing adjuvant regime. To put the observations of the OlympiA trial into perspective, there is a need to evaluate adjuvant capecitabine benefit in tumours with homologous recombination deficiency (HRD), to which g*BRCA-*mutated tumours belong.

Homologous recombination deficiency may serve as a predictive biomarker to guide decisions on DNA-damaging agents, such as bifunctional alkylating agents, platinum salts and PARP inhibitors, as systemic therapy for patients with early-stage TNBC [13–18]. In unselected TNBC patients, ~10% of the patients harbor a deleterious *BRCA1/2* mutation, which results in tumours that are deficient in homologous recombination [19–23]. In TNBC patients without a germline *BRCA1/2* mutation, a significant number of tumours harbor HRD [14, 24, 25]. The array comparative genomic hybridisation (aCGH) *BRCA1*-like and *BRCA2*-like classifiers are two HRD-tests that have been developed from the characteristic DNA copy number aberrations of *BRCA1*- and *BRCA2*-mutated breast cancers, respectively [26, 27]. The *BRCA1*-like classifier showed clinical validity and utility to predict the benefit of intensified platinum-based chemotherapy for stage III HER2-negative breast cancer patients [15, 28–30] and for stage III TNBC patients [15]. The predictive value of the *BRCA2*-like classifier in TNBC is currently unknown and difficult to evaluate due to the low incidence of TNBC with a *BRCA2*-like phenotype in the absence of a *BRCA1*-like phenotype [28]. Notably, the predictive value of the *BRCA1*-like classifier for outcome after (neo)adjuvant treatment with other DNA damaging agent-containing regimens and/or dose-intensities in TNBC has not been established yet.

We hypothesise that patients with non-*BRCA1*-like tumours are the subgroup of TNBC tumours that receive benefit of the addition of capecitabine to adjuvant standard chemotherapy. Our hypothesis is based on the following observations. First, in a predefined subgroup analysis of the GEICAM-CIBOMA study, the addition of capecitabine to standard (neo)adjuvant chemotherapy resulted in significant DFS and OS improvement in patients with non-basal-like TNBC, but not in those with basal-like phenotypes [31]. Second, capecitabine demonstrated improved outcome in patients with advanced breast cancer pretreated with an anthracycline-based regimen [32] suggesting that capecitabine could be more effective in tumours that have intrinsic or acquired resistance to DNA-damaging regimens. HRD and basal-like tumours seem generally sensitive to regimens containing standard DNA-damaging agents such as anthracyclines and cyclophosphamide [33]. One could hypothesise that the notable improved outcome of the TNBC patients treated with capecitabine in the CREATE-X trial [5], limited to patients with residual disease after neoadjuvant treatment with anthracyclines, taxanes or both, was driven by patients enriched with non-*BRCA1*-like or resistant *BRCA1*-like tumours.

Our aim is to evaluate whether *BRCA1*-like status determines the benefit of adjuvant capecitabine-containing systemic treatment in early-stage TNBC patients within the FinXX trial. The FinXX trial is a large phase III, randomised controlled trial comparing adjuvant conventional chemotherapy with adjuvant capecitabine-containing chemotherapy [7].

## Methods

### Patients

We studied early-stage TNBC patients who were included in the Finland Capecitabine (FinXX) trial (NCT00114816); a large, multicenter, randomised controlled clinical trial conducted in Finland and Sweden between 2004 and 2007 [7, 34]. Eligibility criteria have been published previously [7]. In summary, patients were younger than 65 years, had histologically confirmed invasive breast cancer with either regional lymph nodes containing cancer or node-negative cancer with primary tumours of ≥20 mm diameter and negative progesterone receptor (PR) expression in immunohistochemistry, no distant metastases and no prior neoadjuvant chemotherapy. TNBC was defined as estrogen (ER) and progesterone receptor (PR) negativity (<10%), and no HER2 overexpression (determined either by immunohistochemistry or in situ hybridisation). The study was approved by the Ethics Committee of the participating medical institutions and the National Agency for Medicines, Finland. Patients supplied written informed consent to allow the use of their tumour tissue for clinical study-related research purposes. The Institutional Review Board at the Helsinki University Hospital, Finland, approved the use of archival tissue for the current translational study.

### Treatment

Patients were randomised in a 1:1 ratio to either an adjuvant capecitabine(X)-containing chemotherapy regimen (TX + CEX: 3 cycles of capecitabine 900 mg/m² twice daily on day 1–15 plus docetaxel 60 mg/m² 3-weekly, followed by 3 cycles of cyclophosphamide 600 mg/m², epirubicin 75 mg/m² and capecitabine 900 mg/m² twice daily on day 1–15, 3-weekly) or to adjuvant conventional chemotherapy (T + CEF: 3 cycles of docetaxel 80 mg/m² 3-weekly, followed by 3 cycles of cyclophosphamide 600 mg/m², epirubicin 75 mg/m² and fluorouracil 600 mg/m², 3-weekly). Patients received locoregional radiotherapy after completion of chemotherapy according to the local guidelines.

### DNA extraction

Tumour DNA was isolated from two 10 µm whole slides of formalin-fixed paraffin-embedded (FFPE) tissue containing at least 50% tumour cells. Manual microdissection was carried out for slides containing ≤50% of representative tumour area to increase the percentage of neoplastic cells. Paraffin was removed with Qiagen’s Deparaffinization Solution, and tissue was lysed using a mixture of 20 μL Proteinase K (20 mg/ml, included in the QIAsymphony DSP DNA kit) and 200 μL lysis buffer (0.05 M Tris-HCl ph 8.5, 0.04 mM EDTA, 0.5% Tween20) per sample at 56 °C overnight. DNA extraction was performed with QIAsymphony SP instrument using DSP DNA mini kit with 100 µL elution volume (Qiagen, Venlo, The Netherlands).

### Low-coverage whole genome sequencing and data processing

The amount of double-stranded DNA in the genomic DNA samples was quantified using the Qubit® dsDNA HS Assay Kit (Invitrogen, cat no Q32851). Up to 500 ng of double-stranded genomic DNA was fragmented using ultrasonicator shearing (Covaris.com, Massachusetts, USA) to obtain fragment sizes of 160–180 bp. Samples were purified using 1.8X Agencourt AMPure XP PCR Purification beads according to the manufacturer’s instructions (Beckman Coulter, cat no A63881). DNA library preparation for Illumina sequencing was performed using the KAPA Hyper Prep Kit (KAPA Biosystems, KK8504). During the ligation 144 unique adapter indices, manufactured by IDT (Integrated DNA Technologies IDT, Inc. Coralville, Iowa, USA), were used in a molarity of 15 µM. Six PCR cycles were used during library enrichment to obtain enough yield for sequencing. All DNA libraries were analysed on the Caliper GX bioanalyzer (PerkinElmer) using the HT DNA High Sensitivity LabChip for determining the molarity. Up to 133 uniquely indexed samples were mixed together by equimolar pooling. The pools were analysed on the Agilent Technologies 2100 Bioanalyzer and subsequently diluted to 10 nM. Each pool was subjected to sequencing in one lane of a single read 65 bp run, on an Illumina HiSeq2500 machine, according to the manufacturer’s instructions.

Reads were aligned to the reference genome GRCh38 using BWA-MEM algorithm (version 0.7.17) [35]. Per bin of 20 kb, using BEDTools [36], reads on autosomes were counted. Excluded were sites attracting excessive anomalous read mappings (ENCODE) [37] and bins that had a GRCh38 reference mappability below 0.2. Mappability is the fraction of 65 bp sequences, per bin, that aligns to itself. Local GC effects in samples were fitted with non-linear loess, including a subset of reference mappabilities over 0.8, to correct sample bin counts. A line can be fitted through the origin and center of GC corrected counts per mappability density. The slope of this line is used to scale mappabilities to reference counts. Genomic profiles consist of log2 ratios of GC corrected bin counts divided by these scaled reference counts. The sequencing data discussed in this publication have been deposited in NCBI’s Sequence Read Archive (SRA) and are accessible through BioProject number PRJNA647428 [38].

### *BRCA1*-like classification

Genomic profiles were analysed using the *BRCA1*-like classifier, which was originally developed using array comparative genomic hybridisation (aCGH) data generated from breast cancers that were or were not associated with germline *BRCA1* mutations [26]. In brief, the *BRCA1*-like classifier is a shrunken centroid classifier that assigns a genomic profile to a *BRCA1*-like class using a probability score between 0 (non-*BRCA1*-like) and 1 (*BRCA1*-like). The threshold for assigning a breast tumour to the *BRCA1*-like group was set at ≥0.63 as obtained and validated in previous studies [15, 28–30]. The *BRCA1*-like classifier can be used on genomic copy number variation (CNV) profiles obtained by low-coverage whole genome sequencing [39, 40]. Recently, we implemented several updates in the processing of CNV sequencing (CNVseq) data and validated the *BRCA1*-like classification obtained with these data. A detailed description is provided in the Supplementary information (Supplementary Methods; Supplementary Figs. S1 and S2; Supplementary Tables S1 and S2). The R code of this classifier is available at http://ccb.nki.nl/software/nkibrca/. In brief, the *BRCA1*-like classification of copy number profiles can reliably be obtained with the updated CNVseq data with an accuracy of 85–93% when compared to the original BAC aCGH *BRCA1*-like classifier (which is similar to previously established performance on low-coverage, whole genome next-generation sequencing) [39].

Quality checks of the CNV profiles of the TNBC FinXX patients were performed blinded for *BRCA1*-like score and outcome. Samples with low quality were excluded from analyses.

Previously, the identification of BRCAness has been explored on the same dataset of early-stage TNBC patients using the RNA-based NanoString BRCAness signature [12]. Signature scores were calculated using prescribed algorithms developed by NanoString technologies [41]. In the present study, we additionally compared the concordance of our DNA-based CNV *BRCA1*-like classifier with the RNA-based NanoString BRCAness signature. Furthermore, patient-level data regarding PAM50-intrinsic subtype was available for 111 TNBC patients of the FinXX trial [12] and used to classify non-*BRCA1*-like and *BRCA1*-like patients into the Luminal A, Luminal B, HER2-enriched or basal-like subtype.

### Statistical analysis

Characteristics of patients were compared by *BRCA1*-like status using Fisher’s exact, chi-square or linear-by-linear tests for categorical variables and Mann-Whitney U tests for continuous variables.

Recurrence-free survival (RFS) was defined as the time from randomisation to local or distant invasive breast cancer recurrence, death from any cause, or to the last date of follow-up, whichever occurred first. Overall survival (OS) was defined as the time from randomisation to death from any cause or the last date of follow-up. Median follow-up was calculated using the reverse Kaplan-Meier estimator. Survival curves were computed with the Kaplan-Meier method. To evaluate whether the benefit from adjuvant capecitabine-containing chemotherapy versus adjuvant conventional chemotherapy differs between *BRCA1*-like and non-*BRCA1*-like tumours, we applied Cox proportional hazards regression with an interaction term between treatment and *BRCA1*-like status. We estimated and compared interaction coefficients that were unadjusted and adjusted for the following variables: age at randomisation, World Health Organization (WHO) performance status (0, 1), type of surgery (breast-conserving, mastectomy), axillary surgery (dissection, sentinel node biopsy), T-stage (pT1, pT2, pT3), axillary nodal status (≤3 vs >3 positive lymph nodes), histological type (ductal, lobular, other) and histological grade (1, 2, 3). Due to the relatively small number of events, interaction coefficients were adjusted for one covariate at a time. The prognostic effects of all covariates were also evaluated in separate models. The proportionality of hazards was checked using Schoenfeld residuals. A two-sided *p*-value <0.05 was considered statistically significant. Statistical analyses were performed using SPSS version 25 (IBM Corp., Armonk, NY, USA) and Stata 16 (StataCorp. 2019. College Station, TX, USA).

To determine the concordance between our DNA-based CNV *BRCA1*-like classifier and the RNA-based NanoString BRCAness signature, we dichotomised the acquired continuous scores at the percentile of the established cut-off for the *BRCA1*-like classifier, as there is no predefined cut-off for the NanoString BRCAness signature.

## Results

### Patient characteristics

**Fig. 1:**
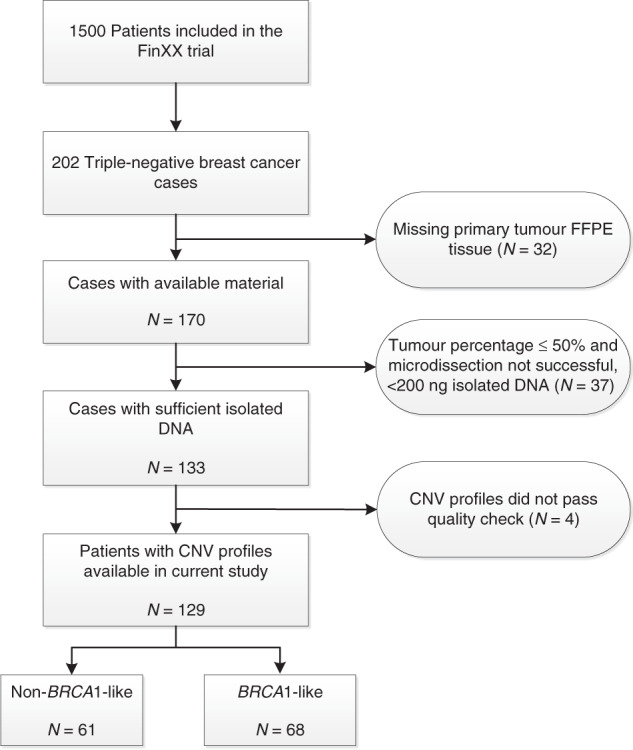
Flow diagram of patient selection in the current study. Reasons for dropout are listed. Tumours of 129 patients could be evaluated for *BRCA1*-like status. Triple-negative breast cancer was defined as estrogen (ER) and progesterone receptor (PR) negativity (<10%), and no HER2 overexpression. FFPE formalin-fixed paraffin-embedded, CNV copy number variation, *BRCA1*-like *BRCA1*-like profile based on low-coverage whole genome DNA next-generation sequencing (lcNGS). Non-*BRCA1*-like no *BRCA1*-like profile based on lcNGS.

Of 202 TNBC patients included in the FinXX trial, we obtained *BRCA1*-like status for 129 (63.9%) patients (Fig. 1). The main reasons for failure were lack of available tumour tissue, low tumour percentage and insufficient amounts of isolated DNA. This subgroup of 129 TNBC patients did not differ substantially for the variables mentioned in Table 1 from those TNBC FinXX patients not included in the current analyses (Supplementary Table S3). Sixty-one (47.3%) of the 129 tumours had a non-*BRCA1*-like profile (Table 1). As expected, patients with a non-*BRCA1*-like tumour had less frequently poorly differentiated tumours compared to patients with *BRCA1*-like tumours (*P* = 0.03) and had significantly more often more than three positive axillary lymph nodes (*P* = 0.047). Furthermore, non-*BRCA1*-like tumours had a lower T-stage (*P* = 0.03) and almost half were classified into the non-basal-like subtype. All *BRCA1*-like tumours for which PAM50-intrinsic subtype data were available, were classified into the basal-like subtype.

**Table 1:** Characteristics of TNBC patients with known *BRCA1*-like status.

Characteristic	Total		Patients with a *BRCA1*-like profile		Patients with a non-*BRCA1*-like profile		*P*-value
	*N*	(%)	*N*	(%)	*N*	(%)	
Total	129	(100)	68	(52.7)	61	(47.3)	
Median (IQR) age at study entry, y	53	(45–59)	52	(44–58)	54	(48–60)	0.11
WHO performance status							0.82
0	109	(84.5)	57	(83.8)	52	(85.2)	
1	20	(15.5)	11	(16.2)	9	(14.8)	
Median (IQR) tumour diameter, mm	25	(21–35)	28	(22–35)	25	(19–35)	0.07
T-stage							0.03
pT1	32	(24.8)	11	(16.2)	21	(34.4)	
pT2	87	(67.4)	51	(75.0)	36	(59.0)	
pT3	10	(7.8)	6	(8.8)	4	(6.6)	
Histological grade							0.03
1	1	(0.8)	0	(0)	1	(1.6)	
2	15	(11.6)	4	(5.9)	11	(18.0)	
3	113	(87.6)	64	(94.1)	49	(80.3)	
Histological type							0.48
Ductal	120	(93.0)	65	(95.6)	55	(90.2)	
Lobular	3	(2.3)	1	(1.5)	2	(3.3)	
Other	6	(4.7)	2	(2.9)	4	(6.6)	
PAM50-intrinsic subtype							<0.001
Luminal A	5	(3.9)	0	(0)	5	(8.2)	
Luminal B	3	(2.3)	0	(0)	3	(4.9)	
HER2-enriched	13	(10.1)	0	(0)	13	(21.3)	
Basal-like	80	(62.0)	58	(85.3)	22	(36.1)	
Unknown	28	(21.7)	10	(14.7)	18	(29.5)	
Axillary nodal status							0.047
≤3	97	(75.2)	56	(82.4)	41	(67.2)	
>3	32	(24.8)	12	(17.6)	20	(32.8)	
Type of surgery							0.53
Breast-conserving	43	(33.3)	21	(30.9)	22	(36.1)	
Mastectomy	86	(66.7)	47	(69.1)	39	(63.9)	
Axillary surgery							0.02
Dissection	111	(86.0)	54	(79.4)	57	(93.4)	
Sentinel node biopsy	18	(14.0)	14	(20.6)	4	(6.6)	
Treatment							0.82
T + CEF	69	(53.5)	37	(54.4)	32	(52.5)	
TX + CEX	60	(46.5)	31	(45.6)	29	(47.5)	

*P*-values: patients with a *BRCA1*-like profile were compared with patients with a non-*BRCA1*-like profile. *P*-values were calculated using Fisher’s exact, chi-square or linear-by-linear tests for categorical variables and Mann–Whitney U tests for continuous variables. Patients with unknown values were omitted.

*TNBC* triple-negative breast cancer, *BRCA1*-like *BRCA1*-like profile based on low-coverage whole genome DNA next-generation sequencing (lcNGS). Non-*BRCA1*-like no *BRCA1*-like profile based on lcNGS, IQR interquartile range, WHO World Health Organization, T + CEF 3 cycles of docetaxel 3-weekly, followed by 3 cycles of cyclophosphamide, epirubicin and fluorouracil, 3-weekly, TX + CEX 3 cycles of capecitabine plus docetaxel 3-weekly, followed by 3 cycles of cyclophosphamide, epirubicin and capecitabine, 3-weekly.

### Association of non-*BRCA1*-like status with survival

The median follow-up was 10.7 years for all 129 patients, with 35 recurrences and 32 deaths, and with a total person-time of 1085 years. In this cohort, non-*BRCA1*-like status was not significantly associated with prognosis: the unadjusted hazard ratios (HRs) of RFS and OS for non-*BRCA1*-like patients when compared to *BRCA1*-like patients were 1.35 (95% CI 0.69–2.63) and 1.27 (95% CI 0.64–2.56), respectively (Table 2). A high number (>3) of positive lymph nodes was significantly associated with an unfavourable RFS (HR 2.13; 95% CI 1.07–4.22), whereas T-stage (pT3 versus pT1 or pT2: HR 1.90; 95% CI 0.67–5.39) and histological grade was not (grade 3 versus grade 1 or 2: HR 0.79; 95% CI 0.31–2.04).

**Table 2:** Cox proportional hazards analyses of the prognostic and predictive value of *BRCA1*-like status for RFS and OS.

	RFS				OS			
Variable	No. events/no. patients	HR^a^	95% CI	*P*-value	No. events/no. patients	HR^a^	95% CI	*P*-value
DNA-based CNV pattern								
* BRCA1*-like	19/61	1			17/61	1		
Non-*BRCA1*-like	16/68	1.35	0.69–2.63	0.37	15/68	1.27	0.64–2.56	0.49
Non-*BRCA1*-like tumours								
T + CEF	15/32	1			14/32	1		
TX + CEX	4/29	0.23^b^	0.08–0.70	<0.01	3/29	0.19^c^	0.05–0.66	<0.01
*BRCA1*-like tumours								
T + CEF	10/37	1			9/37	1		
TX + CEX	6/31	0.66^b^	0.24–1.81	0.42	6/31	0.75^c^	0.27–2.11	0.59

*RFS* recurrence-free survival, *OS* overall survival, *HR* hazard ratio, *CI* confidence interval, *CNV* copy number variation, T + CEF 3 cycles of docetaxel 3-weekly, followed by 3 cycles of cyclophosphamide, epirubicin and fluorouracil, 3-weekly, TX + CEX 3 cycles of capecitabine plus docetaxel 3-weekly, followed by 3 cycles of cyclophosphamide, epirubicin and capecitabine, 3-weekly.

Interaction test between *BRCA1*-like status and chemotherapy regimen:

^a^All Cox proportional hazard analyses shown here were unadjusted for clinicopathologic variables. Similar results were obtained when adjusted for one covariate at the time (due to the relatively small number of events).

^b^*P* = 0.17;

^c^*P* = 0.09.

### Association of non-*BRCA1*-like status with the benefit of adjuvant capecitabine-containing chemotherapy

**Fig. 2:**
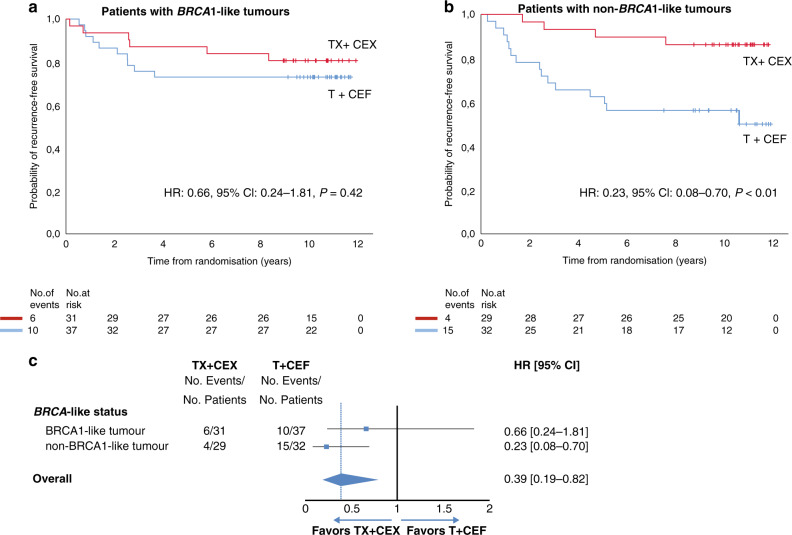
Recurrence-free survival for TNBC patients by *BRCA1*-like status and allocated adjuvant treatment. Kaplan–Meier curves of RFS for TNBC patients with *BRCA1*-like (**a**) and non-*BRCA1*-like tumours (**b**) according to treatment. Number of events and patients at risk are reported below the figure. Unadjusted hazard ratios are derived from Cox regression models (**a**, **b**). Similar results were obtained when HRs were adjusted for clinicopathologic variables. **c** Forest plot of hazard ratios for recurrence-free survival according to *BRCA1*-like status and treatment. Patients had been randomly assigned between adjuvant TX + CEX or T + CEF. HR hazard ratio, CI confidence interval, RFS recurrence-free survival, TNBC triple-negative breast cancer, TX + CEX 3 cycles of capecitabine plus docetaxel 3-weekly, followed by 3 cycles of cyclophosphamide, epirubicin and capecitabine, 3-weekly, T + CEF 3 cycles of docetaxel 3-weekly, followed by 3 cycles of cyclophosphamide, epirubicin and fluorouracil, 3-weekly.

Overall, adjuvant capecitabine-containing chemotherapy (TX + CEX) was more effective than the conventional chemotherapy (T + CEF) in our cohort of 129 TNBC patients (RFS: HR 0.39; 95% CI 0.19–0.82; *P* = 0.01). This is in line with the treatment effect in all 202 TNBC cases of the FinXX trial (RFS: HR 0.54; 95% CI 0.31–0.92; *P* = 0.02) [34]. While in non-*BRCA1*-like patients adjuvant capecitabine-containing chemotherapy was significantly more effective than conventional chemotherapy (RFS: HR 0.23; 95% CI 0.08–0.70; *P* < 0.01), this was not observed in patients with a *BRCA1*-like tumour (RFS: HR 0.66; 95% CI 0.24–1.81; *P* = 0.42). However, the beneficial effect of the adjuvant capecitabine-containing regimen did not differ significantly by *BRCA1*-like status (P interaction = 0.17) (Table 2, Fig. 2). Similar results were obtained after adjustment for each of the clinicopathologic variables (*P*-values ranging from 0.15 to 0.22). Furthermore, similar results were observed for OS (P interaction = 0.09) (Table 2, Supplementary Fig. S3).

### DNA-based CNV *BRCA1*-like status versus RNA-based NanoString BRCAness signature

**Fig. 3:**
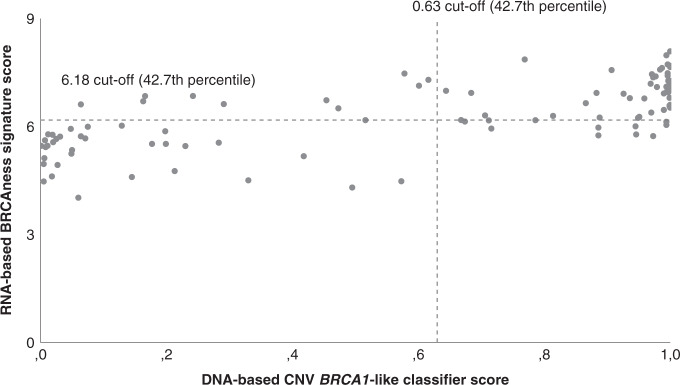
DNA-based CNV *BRCA1*-like classifier versus RNA-based BRCAness signature. Scatterplot illustrating the corresponding DNA-based CNV *BRCA1*-like classifier score and RNA-based NanoString BRCAness signature score belonging to the same tumour. Both scores were available for 103/202 TNBC patients. The dashed lines illustrate the cut-off points when the scores are dichotomised at the percentile of the established cutoff for the *BRCA1*-like classifier (42.7th percentile).

Both scores of DNA-based CNV *BRCA1*-like classifier and the RNA-based NanoString BRCAness signature were available for 103/202 TNBC patients (Fig. 3). The established cutoff for the *BRCA1*-like classifier (0.63) occurred at the 42.7th percentile in this dataset. Since the BRCAness signature score does not have an established cut-off, we dichotomised at the 42.7th percentile of the ranked BRCAness scores (6.18). For 78.6% (81/103) there is concordance in *BRCA1*-like/BRCAness classification. The 21.4% (22/103) disagreement is equal in both directions and the discordant patients are at intermediate risk of recurrence between the concordant patients.

## Discussion

In the present study we observed a significant and pronounced benefit with the addition of capecitabine to adjuvant conventional chemotherapy for patients with non-*BRCA1*-like TNBC. The data were less conclusive regarding the benefit for the *BRCA1*-like group, possibly due to the size of the trial and power limitations, and, therefore, we cannot exclude the hypothesis that the addition of capecitabine to conventional chemotherapy benefits also patients with *BRCA1*-like TNBC. The large benefit in non-*BRCA1*-like patients appears to justify offering these patients adjuvant treatment that includes capecitabine.

Our observations may be explained by the mechanism of action of capecitabine. Although capecitabine causes DNA damage [11], it may not specifically result in DNA damage that is dependent on proficient homologous recombination machinery resulting in error-free DNA repair [42]. In fact, antimetabolites such as capecitabine and its active form 5-fluorouracil lead to (1) DNA base pair mismatches which are repaired by the DNA mismatch repair (MMR) pathway [43], and (2) inhibition of DNA replication, leading to abasic sites that are repaired by base excision repair (BER) proteins [44]. Therefore, a *BRCA1*-like profile will most likely not yield enhanced sensitivity to treatment with capecitabine.

Our observation of the benefit of capecitabine in patients with non-*BRCA1*-like tumours is in line with Alli et al. who found a 5-fold higher sensitivity to 5-fluorouracil of wild-type *BRCA1* compared to *BRCA1*-deficient murine mammary epithelial cells [45]. We did, however, not find a predictive value of the *BRCA1*-like status, as is consistent with preclinical findings of Quinn et al. who observed no differential dose-response effect of capecitabine in *BRCA1*-mutated compared with wild-type *BRCA1* human BC cells [46]. Currently, the NordicTrip (ClinicalTrials.gov Identifier: NCT04335669) is an ongoing translational clinical trial in early-stage TNBC patients prospectively comparing the effect of adding capecitabine to neoadjuvant epirubicin plus cyclophosphamide followed by carboplatin plus paclitaxel on pathologic complete response (pCR) rate, stratified for HRD positive versus HRD negative/ HRD-intermediate. Results of this study have to be awaited to further clarify the value of HRD as a predictive biomarker for benefit of capecitabine-containing chemotherapy in early-stage TNBC.

Non-basal triple-negative breast tumours seem particularly sensitive to the addition of capecitabine to standard (neo)adjuvant chemotherapy, as has been demonstrated in the ECOG-ACRIN EA1131 trial and from a pre-defined subgroup analysis of the GEICAM-CIBOMA trial [31, 47]. Almost half of the non-*BRCA1*-like TNBC have a non-basal-like profile, in contrast to the *BRCA1*-like TNBC of which the great majority has a basal-like profile, as was previously observed [15, 48] and confirmed in the current study. Hence, our observations of a pronounced benefit of adjuvant capecitabine in non-*BRCA1*-like TNBC patients is in accordance with the increased sensitivity of adjuvant capecitabine in non-basal-like TNBC.

In our studied TNBC patients, 61% had a *BRCA1*-like tumour. In general, the proportion of TNBC patients with a *BRCA*-like/ BRCAness tumour depends on the patient case-mix and the method used to identify HRD. In the present study we included patients that fulfilled the selection criteria of the FinXX trial and had available tumour material that was of sufficient quality to successfully generate a *BRCA1*-like test result. These patients may therefore not be representative of the general TNBC population. However, the observed proportion of triple-negative *BRCA1*-like tumours is in concordance with earlier observations where the same classifier has been used [28, 49].

The main strength of our study is the study design, i.e., a prospective, randomised controlled trial with collection of archival material. This prospective-retrospective design is the first choice to assess a putative predictive biomarker in case a prospective randomised clinical trial is not feasible, because such trials require huge numbers of patients, are costly and take many years to complete [50]. An additional strength, in contrast to the exploratory analyses of Asleh et al. using an RNA 800-gene panel without predefined cutoff for the BRCAness signature [12], is that we evaluated a single biomarker with a predefined cutoff based on prior biological and empirical evidence and with a clear hypothesis [26, 28–30]. Such an approach is required to establish the implementation of a predictive biomarker in clinical practice, or to refute it [50].

A limitation of the present study is the small sample size with few events, which is due to the fact that the FinXX trial was powered to evaluate the main effect of capecitabine among patients with any molecular subtype of breast cancer rather than a treatment-marker interaction in the subgroup of TNBC patients. In addition, the number of patients was further reduced by the failure to obtain *BRCA1*-like status for all TNBC patients for several reasons. The small sample size might explain why we did not observe a significant interaction between *BRCA1*-like status and capecitabine-containing chemotherapy in these unplanned subgroup analyses. However, our patient group accounts for 129 (63.9%) of the 202 accrued TNBC patients in the FinXX trial, which is within the recommended range of sample size for a study to evaluate predictive biomarkers [50]. Furthermore, the included patients did not differ substantially from all accrued TNBC patients for the evaluated clinical variables and outcomes. An additional limitation is that a small diluting effect of fluorouracil in the control arm on the effect of adjuvant capecitabine in the intervention arm cannot be excluded. However, this effect is expected to not substantially influence the observed results of our analyses in *BRCA1*-like versus non-*BRCA1*-like TNBC since there are two main differences between the treatment arms. First, the cumulative exposure to fluorouracil in the control arm, given as a single infusion on day 1 of each 3-week cycle (3 cycles in total), is substantially lower than the cumulative exposure to capecitabine in the intervention arm, which is given twice daily on days 1–15 of each 3-week cycle (6 cycles in total). Secondly, oral capecitabine on consecutive days resembles a continuous infusion of fluorouracil, which represents a more effective therapy compared to a single bolus infusion of fluorouracil [51]. The third limitation is that our study did not address whether *BRCA1*-like status has predictive value for capecitabine-containing chemotherapy only in individuals with residual disease after neoadjuvant chemotherapy. Collaborative efforts to further elucidate this in the CREATE-X trial are ongoing.

Our findings may have implications for treatment decisions in early-stage TNBC patients. Currently, the addition of PARP inhibitors in the (neo)adjuvant treatment of early-stage TNBC patients is an emerging area of investigation [16, 52–54]. The OlympiA trial is a pivotal trial that evaluated the efficacy of adjuvant treatment with olaparib, a PARP inhibitor, compared to placebo in patients with non-metastatic, germline *BRCA* mutated (*gBRCAm*), high risk, HER2-negative primary breast cancer [13]. Adjuvant olaparib resulted in 42% risk reduction of IDFS events at 3 years compared to placebo. However, since these patients were not treated according to current clinical practice with adjuvant capecitabine, the additional benefit of olaparib to capecitabine is unclear. In our present study evaluating the benefit of adjuvant capecitabine, the HR of 0.66 for RFS in *BRCA1*-like TNBC patients is indicative of a clinical benefit of the addition of capecitabine in tumours with HRD, although no statistical significance was achieved. Further research evaluating the efficacy of the combination of olaparib and capecitabine as compared to only one of these drugs is needed. Additionally, not all *gBRCAm* tumours have dysfunctional homologous recombination (HR), for instance due to the type of *BRCA1* mutation [55] or restored HR by genetic reversion of the underlying BRCA mutation [56–58], and could biologically be considered as homologous recombination proficient. It will be of interest to explore if the *gBRCAm* patients of the OlympiA trial that did not benefit from olaparib are the ones with functional HR. Based on our results, these patients should be treated with capecitabine.

In conclusion, the addition of capecitabine to standard adjuvant chemotherapy appears to improve RFS in patients with non-*BRCA1*-like TNBC. Patients with *BRCA1*-like TNBC may also benefit, but this did not reach statistical significance. Therefore, it is now important to investigate the *BRCA1*-like status in other series that have evaluated adjuvant capecitabine in the treatment of early-stage TNBC. Furthermore, additional research is needed to identify a biomarker that upfront defines the subgroup within the *BRCA1*-like TNBC patients who do not benefit.

## Supplementary information

Supplementary Information

REMARK guidelines

Academic Journals Reporting Checklist BJC-A3338268

## Data Availability

The clinical datasets used and analysed during the current study are available from Heikki Joensuu on reasonable request. The sequencing datasets generated and analysed during the current study are available in the SRA repository, [https://www.ncbi.nlm.nih.gov/Traces/study1/?acc=PRJNA647428&].

## Code availability

The R code to produce the *BRCA1*-like classification presented in this manuscript is available at http://ccb.nki.nl/software/nkibrca/. The full code is available upon reasonable request.
