# Supplementary material for: Adjuvant capecitabine-containing chemotherapy benefit and homologous recombination deficiency in early-stage triple-negative breast cancer patients
Source: Br J Cancer. 2022 Feb 5;126(10):1401–9. doi: 10.1038/s41416-022-01711-y (PMC9090783; doi:10.1038/s41416-022-01711-y)
Supplement: Supplementary file 1 — Supplementary Information [file 41416_2022_1711_MOESM1_ESM.docx]

**Adjuvant capecitabine-containing chemotherapy benefit and homologous recombination deficiency in early-stage triple-negative breast cancer patients**

Leonora W. de Boo, Katarzyna Jóźwiak, Heikki Joensuu, Henrik Lindman, Susanna Lauttia, Mark Opdam, Charlaine van Steenis, Wim Brugman, Roelof J.C. Kluin, Philip C. Schouten, Marleen Kok, Petra M. Nederlof, Michael Hauptmann, Sabine C. Linn

**Supplementary information**

Contents

[Supplementary Methods 2](#_Toc92824218)

[Figure S1 5](#_Toc92824219)

[Figure S2 6](#_Toc92824220)

[Table S1 7](#_Toc92824221)

[Table S2 8](#_Toc92824222)

[Table S3 9](#_Toc92824223)

[Figure S3 10](#_Toc92824224)

## Supplementary Methods

**Supplementary Methods: Detailed method of the update and validation of the low coverage CNV sequencing *BRCA1*-like classifier**

The *BRCA1*-like classifier was developed on copy number (CN) profiles obtained with a bacterial artificial genome (BAC) array Comparative Genomic Hybridization (aCGH) platform [1]. The *BRCA1*-like classifier is a shrunken centroid classifier based on 371 (out of 3277) BAC clones, with a validated threshold at ≥0.63 to assign a breast tumor to the *BRCA1*-like group [2]. In 2015, Schouten et al. validated that the *BRCA1*-like classification of copy number profiles could be obtained reliably with data acquired across multiple platforms. One of these platforms was low coverage copy number variation sequencing (CNVseq) [3]. Since this validation, however, there have been several updates available in the processing of CNVseq data:

- alignment software (BWA-MEM 0.7.17 instead of BWA bt 0.5.10)
- genome version (GRCh38 instead of GRCh37)
- DNA quantification methods (Nanodrop and Qubit PCR in addition to Bioanalyzer)
- longer sequencing read length (65 base pare instead of 51)

Furthermore, lowering the thresholds for mapping quality of the reads (from Q37 into Q15) can increase counts and improve profiles.

Therefore, we investigated whether implementing these changes would affect the *BRCA1*-like classification of CN profiles obtained with CNV sequencing data. We did this by re-analyzing previous datasets, with data generated on both the BAC aCGH and updated CNVseq platform per patient, to evaluate:

1. The similarity of the profiles acquired with the updated CNVseq data and original BAC aCGH data
2. The classification of samples as *BRCA1*-like or non-*BRCA1*-like acquired with the updated CNVseq data and BAC aCGH data

1. The comparison of the profiles acquired with the updated CNV sequencing data and original BAC array CGH data

To evaluate the similarities of the profiles acquired with the updated CNVseq data and BAC aCGH data, we compared the profiles of tumors that were previously repeatedly analyzed on BAC aCGH and CNVseq platforms [3]. Specifically, the subset of patients used for finding optimal parameters was restricted to the patient samples from the Dutch high-dose chemotherapy trial [4].

The update of the alignment software package version (BWA-MEM 0.7.17 instead of BWA bt 0.5.10) and genome version (GRCh38 instead of GRCh37) required us to recreate mappability. Mappability is the fraction of reference genome sections that aligns back to itself. Mappability can provide an expected normal estimate, to create ratios against, for samples processed similarly. Alignment software, sequence length, filters for qualifying reads and windows on the genome wherein sequences are counted need to be identical for samples and commensurate reference genome derived sequences. The mappability was recreated to suit the updated alignment conditions of the BWA-MEM 0.7.17 alignment software. We investigated log(mappability 2014/mappability 2020) profiles and overlapped them with the BAC clones, all lifted to GRCh38, to identify regions that were strongly affected by this change.

One of the updates in the processing of CNVseq data concerned the used genome build version. The GRCh37 and GRCh38 blacklists obtained from ENCODE [5] seemed to overlap only partially. Since the GRCh37 blacklist was already validated, we investigated lifting the GRCh37 blacklist only and merging this lifted blacklist with Encode’s GRCh38 blacklisted regions. We observed that excluding only the lifted GRCh37 blacklist regions resulted in recurrent peaks in the aggregate copy number profile which were not present when excluding regions from the merged blacklist (Supplementary Figure S1). Therefore, we decided to use the merged blacklist.

Subsequently, we observed that the updated CNVseq data results in more pronounced dynamic range of the ratios (gains and losses) compared to the BAC aCGH data. Although we previously also observed that newer and higher resolution platforms showed more pronounced dynamic range (height of the gains and losses) compared to BAC aCGH (which is exemplified by differences in scale of the genomic aberrations) [3], the effect seemed more pronounced with the current updated software. Previously, acceptable concordance was obtained when not correcting for this difference. However, in the current analysis, we obtained again an increase in scale, leading to increased BRCA1-like status misclassification compared to classification acquired with BAC aCGH data. Therefore, we implemented a cross platform correction of scaling and centering of the data. The scaling and centering of the BAC aCGH and updated CNVseq data were recalibrated by using a method similar to quantile normalization. Briefly, we fitted a linear regression model in the statistical software program R with the glm function with Gaussian distribution and identity link function to the sorted genomic position-wise mean values of the BAC aCGH data and the sorted genomic position-wise mean of the new dataset. Subsequently, we used the obtained alpha coefficient to correct the centering and the obtained beta coefficient to correct the scaling.

Steps of the cross platform correction:

1. Calculate the genomic position-wise mean 2log ratio of the mapped CNVseq data over all patients
2. Sort the genomic position-wise mean 2log ratio of the mapped CNVseq data
3. Calculate the genomic position- wise mean of the BAC aCGH data
4. Sort the genomic position-wise mean of the BAC aCGH data
5. Fit a linear regression model between the sorted average BAC array versus sorted CNVSeq data
6. Multiply the CNVSeq data with the beta coefficient thereof and add the alpha coefficient to scale and shift the CNVseq data to BAC data levels

By applying our platform correction, the aggregate CNVseq profile became more similar to the aggregate BAC profile (Supplementary Figure S2; Supplementary Table S1). Furthermore, this resulted in improved similarity between the updated CNVseq pipeline (2020) and the previously used CNVseq pipeline (2015) [3] (Supplementary Table S1).

2. The concordance between the *BRCA1*-like classification acquired with the updated CNVseq data and acquired with the gold standard BAC aCGH classification

Next, we tested whether the updated CNVseq profiles resulted in similar classification as the BAC aCGH profiles, which was considered the gold standard. We therefore compared the tumor *BRCA1*-like classification of two series of breast cancer (BC) specimens for which CN profiles had been generated by both platforms: a set of 93 BC samples, from the Dutch high-dose chemotherapy trial [3, 4], and a set of 76 BC samples, used for the development of the original BAC *BRCA1*-like classifier [1]. The *BRCA1*-like classification based on the updated CNVseq data resulted in an accuracy of 85-93% when compared to the classification based on the BAC aCGH data (Supplementary Table S2 A and B). Most misclassified samples were classified as *BRCA1*-like instead of non-*BRCA1*-like.

In 2015, we performed extensive analysis to identify why some samples are classified differently using the BAC aCGH and CNVseq (2015) platforms [3]. We concluded that the samples that switched class were near the decision boundary or had a low quality BAC aCGH profile. In the present analyses, we used the same CNVseq raw data to produce the updated CNVseq data (2020). We observed that similar classification was reached as in 2015 when we applied the platform correction to the CNVseq (2020) data, with the same samples changing class as we saw before. Furthermore, the discordant classification of the *BRCA1*-like status, obtained with BAC aCGH versus CNVseq, could be explained by low quality of a BAC aCGH profile and/or that the sample in the centroid did not strongly belong to the *BRCA1*-like or non-*BRCA1*-like class.

In conclusion, we validated that the *BRCA1*-like classification of copy number profiles could reliably be obtained with data acquired with the updated CNVseq platform with platform correction. We showed an accuracy of 85-93% for the updated CNVseq *BRCA1*-like classification using the BAC aCGH based *BRCA1*-like classification as the gold standard, which makes this updated CNVseq *BRCA1*-like classifier suitable for research purposes only.

**References**

1. Joosse SA, van Beers EH, Tielen IH, Horlings H, Peterse JL, Hoogerbrugge N, et al. Prediction of BRCA1-association in hereditary non-BRCA1/2 breast carcinomas with array-CGH. Breast Cancer Res Treat. 2009;116(3):479-89.

2. Vollebergh MA, Lips EH, Nederlof PM, Wessels LF, Schmidt MK, van Beers EH, et al. An aCGH classifier derived from BRCA1-mutated breast cancer and benefit of high-dose platinum-based chemotherapy in HER2-negative breast cancer patients. Ann Oncol. 2011;22(7):1561-70.

3. Schouten PC, Grigoriadis A, Kuilman T, Mirza H, Watkins JA, Cooke SA, et al. Robust BRCA1-like classification of copy number profiles of samples repeated across different datasets and platforms. Mol Oncol. 2015;9(7):1274-86.

4. Rodenhuis S, Bontenbal M, Beex LV, Wagstaff J, Richel DJ, Nooij MA, et al. High-dose chemotherapy with hematopoietic stem-cell rescue for high-risk breast cancer. N Engl J Med. 2003;349(1):7-16.

5. Amemiya HM, Kundaje A, Boyle AP. The ENCODE Blacklist: Identification of Problematic Regions of the Genome. Sci Rep. 2019;9(1):9354.

## Figure S1

**Figure S1: Comparison of aggregate BAC aCGH profile and aggregate CNV sequencing profile with the ENCODE GRCh37 (liftover) blacklist only (top) and with the merged ENCODE GRCh37 (liftover) and GRCh38 blacklist (bottom)**


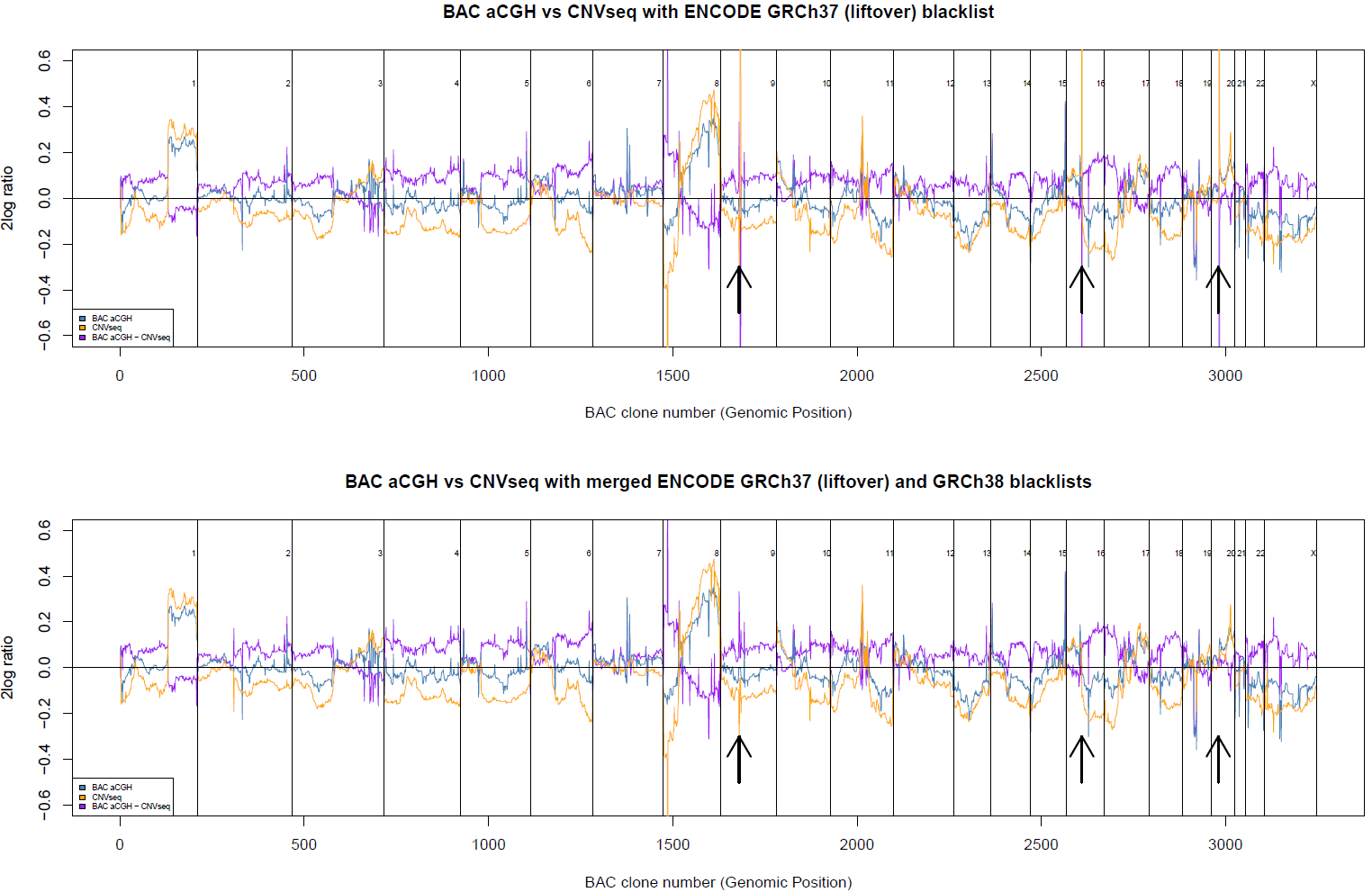


The BAC clone, ordered by genomic position, is shown on the x-axis, and the log2 ratio on the y-axis. The aggregate BAC aCGH profile is plotted in blue, the aggregate CNVseq profile in yellow and the difference in profile between the aggregate BAC aCGH and aggregate CNVseq profile is plotted in purple. The arrows indicate peaks that are removed by using the ENCODE GRCh37 (liftover) blacklist with GRCh38 blacklisted regions. BAC: bacterial artificial genome; aCGH: array Comparative Genomic Hybridization; CNVseq: low coverage copy number variation sequencing.

Figure S2

**Figure S2: Comparison of aggregate BAC aCGH profile and aggregate CNV sequencing profile without (top) and with (bottom) platform correction**


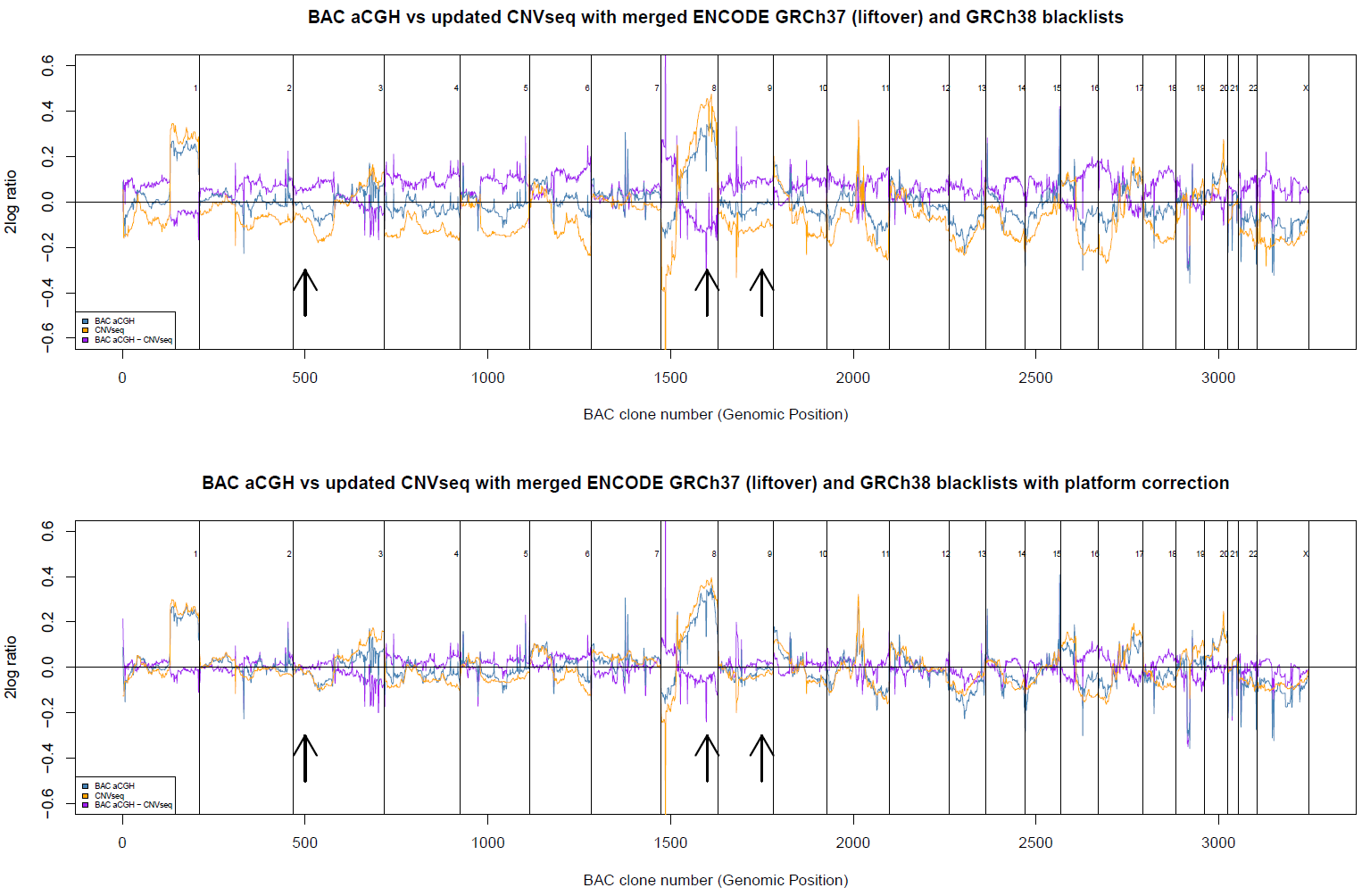


The BAC clone, ordered by genomic position, is shown on the x-axis, and the log2 ratio on the y-axis. The aggregate BAC aCGH profile is plotted in blue, the aggregate CNVseq profile in yellow and the difference in profile between the aggregate BAC aCGH and aggregate CNVseq profile is plotted in purple. The arrows indicate amplifications and deletions that are less pronounced and more similar to the BAC aCGH profile after platform correction of the CNVseq data. BAC: bacterial artificial genome; aCGH: array Comparative Genomic Hybridization; CNVseq: low coverage copy number variation sequencing.

Table S1

**Table S1: Average difference (in log ratios) between the aggregate BAC aCGH and CNVseq profile, with and without platform correction**

| Aggregate profile | Mapped without cross platform correction | Mapped with cross platform correction |
| --- | --- | --- |
| BAC aCGH – CNVseq 2015 | 0.207 | 0.163 |
| BAC aCGH – CNVseq 2020 | 0.158 | 0.120 |

BAC: bacterial artificial genome; aCGH: array Comparative Genomic Hybridization; CNVseq: low coverage copy number variation sequencing. CNVseq 2015: the previously used CNVseq pipeline from 2015. CNVseq 2020: the updated CNVseq pipeline from 2020.

Table S2

**Table S2: The comparison of the classification of samples as BRCA1-like or non-BRCA1-like according to different platforms**

**A**

| **CNVseq platform** | | **BAC aCGH**  **non-*BRCA1*-like** | **BAC aCGH**  ***BRCA1*-like** | **Accuracy** |
| --- | --- | --- | --- | --- |
| **CNVseq 2020**  **without cross platform correction** | non-*BRCA1*-like | 48 | 1 | 0.83 |
|  | *BRCA1*-like | 15 | 29 |  |
| **CNVseq 2020**  **with cross platform correction** | non-*BRCA1*-like | 51 | 2 | 0.85 |
|  | *BRCA1*-like | 12 | 28 |  |
| **CNVseq 2015** | non-*BRCA1*-like | 51 | 0 | 0.87 |
|  | *BRCA1*-like | 12 | 30 |  |

**B**

| **CNVseq platform** | | **BAC aCGH**  **non-*BRCA1*-like** | **BAC aCGH**  ***BRCA1*-like** | **Accuracy** |
| --- | --- | --- | --- | --- |
| **CNVseq 2020**  **without cross platform correction** | non-*BRCA1*-like | 35 | 0 | 0.91 |
|  | *BRCA1*-like | 7 | 34 |  |
| **CNVseq 2020**  **with cross platform correction** | non-*BRCA1*-like | 36 | 0 | 0.93 |
|  | *BRCA1*-like | 5 | 35 |  |

A: a cohort of 93 breast cancer patients from the Dutch high-dose chemotherapy trial.
B: a cohort of 76 breast cancer patients used for the development of the original BAC aCGH *BRCA1*-like classifier.

Accuracy: the ability of the CNVseq platform to reproduce the BAC aCGH classification. CNVseq: low coverage copy number variation sequencing; BAC aCGH: bacterial artificial genome array Comparative Genomic Hybridization (aCGH).

Table S3

**Table S3: Characteristics of the TNBC patients in the studied translational cohort compared to TNBC patients of the original FinXX trial not in the current analyses**

| **Characteristic** | **Total** | | **Patients in the translational study cohort** | | **Patients not in current analyses** | | **P value** |
| --- | --- | --- | --- | --- | --- | --- | --- |
|  | N | (%) | N | (%) | N | (%) |  |
| Total | 202 | (100) | 129 | (63.9) | 73 | (36.1) |  |
| Median (IQR) age at study entry, y | 53 | (45-59) | 53 | (45-59) | 52 | (42-60) | 0.64 |
| WHO performance status |  |  |  |  |  |  | 0.14 |
| 0 | 176 | (87.1) | 109 | (84.5) | 67 | (91.8) |  |
| 1 | 26 | (12.9) | 20 | (15.5) | 6 | (8.2) |  |
| Median (IQR) tumor diameter, mm | 25 | (20-35) | 25 | (21-35) | 25 | (18-37) | 0.46 |
| T-stage |  |  |  |  |  |  | 0.29 |
| pT1 | 55 | (27.2) | 32 | (24.8) | 23 | (31.5) |  |
| pT2 | 133 | (65.8) | 87 | (67.4) | 46 | (63.0) |  |
| pT3 | 14 | (6.9) | 10 | (7.7) | 4 | (5.5) |  |
| Histological grade |  |  |  |  |  |  | 0.13 |
| 1 | 1 | (0.5) | 1 | (0.7) | 0 | (0) |  |
| 2 | 31 | (15.3) | 15 | (11.6) | 16 | (21.9) |  |
| 3 | 170 | (84.2) | 113 | (87.6) | 57 | (78.1) |  |
| Histological type |  |  |  |  |  |  | 0.15 |
| Ductal | 182 | (90.1) | 120 | (93.0) | 62 | (84.9) |  |
| Lobular | 8 | (4.0) | 3 | (2.3) | 5 | (6.8) |  |
| Other | 12 | (5.9) | 6 | (4.7) | 6 | (8.2) |  |
| Axillary nodal status |  |  |  |  |  |  | 0.03 |
| ≤3 | 161 | (79.7) | 97 | (75.2) | 64 | (87.7) |  |
| >3 | 41 | (20.3) | 32 | (24.8) | 9 | (12.3) |  |
| Type of surgery |  |  |  |  |  |  | 0.64 |
| Breast conserving | 65 | (32.2) | 43 | (33.3) | 22 | (30.1) |  |
| Mastectomy | 137 | (67.8) | 86 | (66.7) | 51 | (69.9) |  |
| Axillary surgery |  |  |  |  |  |  | 0.47 |
| Dissection | 171 | (84.7) | 111 | (86.0) | 60 | (82.2) |  |
| Sentinel node biopsy | 31 | (15.3) | 18 | (14.0) | 13 | (17.8) |  |
| Treatment |  |  |  |  |  |  | 0.86 |
| T+CEF | 109 | (54.0) | 69 | (53.5) | 40 | (54.8) |  |
| TX+CEX | 93 | (46.0) | 60 | (46.5) | 33 | (45.2) |  |
| Median RFS (years) /no of events | 10.5 | /59 | 10.7 | /35 | 10.1 | /24 |  |

The great majority of the FinXX trial participants were Caucasian. P values: patients with acquired *BRCA1*-like status were compared with patients not in the current analyses. P values were calculated using Fisher’s exact, chi-square or a linear-by-linear test for categorical variables, and a Mann-Whitney U test for continuous variables.

TNBC: triple negative breast cancer; *BRCA1*-like: *BRCA1*-like profile based on low coverage whole genome DNA next generation sequencing (lcNGS). Non-*BRCA1*-like: no *BRCA1*-like profile based on lcNGS. IQR: interquartile range; WHO: World Health Organization; T+CEF: 3 cycles of docetaxel 3-weekly, followed by 3 cycles of cyclophosphamide, epirubicin, and fluorouracil, 3-weekly; TX+CEX: 3 cycles of capecitabine plus docetaxel 3-weekly, followed by 3 cycles of cyclophosphamide, epirubicin and capecitabine, 3-weekly.

Figure S3

**Figure S3: Overall survival for TNBC patients by BRCA1-like status and adjuvant treatment**

Kaplan-Meier curves of OS for TNBC patients with BRCA1-like (a) and non-BRCA1-like tumors (b) according to treatment.

Patients had been randomly assigned between adjuvant TX+CEX or T+CEF. Number of events and patients at risk are reported below the figure. Unadjusted hazard ratios are derived from Cox regression models. Similar results were obtained when HRs were adjusted for clinico-pathological variables.

HR: hazard ratio; CI: confidence interval; TX+CEX: 3 cycles of capecitabine plus docetaxel 3-weekly, followed by 3 cycles of cyclophosphamide, epirubicin and capecitabine, 3-weekly; T+CEF: 3 cycles of docetaxel 3-weekly, followed by 3 cycles of cyclophosphamide, epirubicin, and fluorouracil, 3-weekly.
